# A New Methodology Based on EMD and Nonlinear Measurements for Sudden Cardiac Death Detection

**DOI:** 10.3390/s20010009

**Published:** 2019-12-18

**Authors:** Olivia Vargas-Lopez, Juan P. Amezquita-Sanchez, J. Jesus De-Santiago-Perez, Jesus R. Rivera-Guillen, Martin Valtierra-Rodriguez, Manuel Toledano-Ayala, Carlos A. Perez-Ramirez

**Affiliations:** 1 ENAP RG, Department of Biomedical Engineering, Faculty of Engineering, Autonomous University of Queretaro, Queretaro 76144, Mexico; ovargas08@alumnos.uaq.mx (O.V.-L.); jamezquita@uaq.mx (J.P.A.-S.); 2 ENAP RG, Department of Electromechanical Engineering, Faculty of Engineering, Autonomous University of Queretaro, San Juan del Rio, Queretaro 76807, Mexico; sapjj@uaq.mx (J.J.D.-S.-P.); jesus.rooney.rivera@uaq.mx (J.R.R.-G.); martin.valtierra@uaq.mx (M.V.-R.); 3 Faculty of Engineering, Autonomous University of Queretaro, Queretaro 76100, Mexico

**Keywords:** EMD, Higuchi fractal value, entropy permutation value, SCD, ECG signal

## Abstract

Heart diseases are among the most common death causes in the population. Particularly, sudden cardiac death (SCD) is the cause of 10% of the deaths around the world. For this reason, it is necessary to develop new methodologies that can predict this event in the earliest possible stage. This work presents a novel methodology to predict when a person can develop an SCD episode before it occurs. It is based on the adroit combination of the empirical mode decomposition, nonlinear measurements, such as the Higuchi fractal and permutation entropy, and a neural network. The obtained results show that the proposed methodology is capable of detecting an SCD episode 25 min before it appears with a 94% accuracy. The main benefits of the proposal are: (1) an improved detection time of 25% compared with previously published works, (2) moderate computational complexity since only two features are used, and (3) it uses the raw ECG without any preprocessing stage, unlike recent previous works.

## 1. Introduction

In the last decade, numerous methodologies or methods have been introduced in the literature for identifying several medical conditions such as diabetes [1,2], glaucoma [3,4], breast cancer [5,6], emotion detection [7], among others. Also, some proposals have been focused on the detection of several neurological disorders such as Alzheimer’s disease (AD) [8,9,10,11], mild cognitive impairment (MCI) [12,13,14,15], delirium [16], epilepsy [17,18,19], sleep disorder [20,21,22], Parkinson’s disease [23,24], autism spectrum disorder [25], schizophrenia [26,27], among others. These methods have been demonstrated to be efficient to diagnose a disease when it has developed symptoms in the human body. For this reason, researchers in the last years, have focused on proposing methodologies or methods capable of predicting disease or detecting it at the earliest possible state. Early identification of disease will allow enrolling the person in treatments or therapies, which can be more effective in an early stage of the disease. In this sense, various researchers have investigated the evolution conversion of MCI to AD [28], prediction of seizure [29], and prediction of sudden cardiac death (SCD) [30,31].

SCD is considered the largest cause of death in society, representing about 10% of deaths around the world [32]. An SCD episode is produced when the heart electrical conductivity changes, generating an inadequate blood circulation to vital organs. This condition puts at risk the patient’s life if the cardiac roots are not timely attended to [33,34]. Different causes such as asystole, ventricular tachycardia, ventricular fibrillation (VF), among others, can produce an SCD episode; but, VF, represents nearly 20% of all SCD events, and is presumed the main cause of an SCD episode [35]. VF is characterized by the electrical activity disorganization in the heart ventricles, inhibiting the ability to efficiently pump blood. It can lead to death in minutes after the onset of symptoms, which is unperceivable to most people [35]. Hence, an early prediction of an SCD event in a patient or person that is suffering a VF is of vital importance because it allows receiving timely treatment and increases the survival chance.

In the last decade, researchers worldwide have presented different methods or methodologies to predict an SCD event using electrocardiogram (ECG) signals as well as the heart rate variability (HRV) [30,31,36,37,38,39,40]. For instance, Murukesan et al. [36] used seven nonlinear features (e.g., number of ectopic beats, among other features) combined with a support vector machine for predicting an SCD event employing HRV of the ECG signals. To validate the proposal, they employ the ECG signals from 18 and 23 patients with normal cardiac rhythm [41] and SCD [42], provided by the database named MIT-BIH, respectively. The authors report an accuracy of 96.36% two minutes before the SCD event. The following works also employ the same databases. Acharya et al., [38] combined six nonlinear features (e.g., approximate entropy, Higuchi fractal dimension, Hurst’s exponent, among other features), with the discrete wavelet transform (DWT), and SVM for predicting an SCD event using the ECG signals. An accuracy of 92.11% 4 min before the onset is reported by the authors. Fujita et al., [39] explored four nonlinear features (fuzzy entropy, Renyi entropy, energy, and Tsallis entropy) and SVM for predicting an SCD event employing HRV of the ECG signals. They report that an accuracy of 94.7% 4 min before the SCD event is reached. Recently, Amezquita-Sanchez et al., [31] investigated the combination of the wavelet packet transform, a nonlinear feature named homogeneity, and an enhanced probabilistic neural network for predicting an SCD episode using ECG signals. The authors report an accuracy of 95.8% 20 min before the onset, improving the window time compared with previous works in which 2, 4, and 12 min before the SCD event were studied [30,36,39]. Additionally, the authors mention that it is necessary to perform further investigation to predict an SCD event much earlier since a larger time window will allow obtaining the medical attention in a timely way to those patients that require to be transported to a hospital. Despite the promising results reported in previous work, it should be noticed that the following aspects can be improved: (1) The amount of computational resources used for the diagnosis, since computationally efficient methodologies can allow real-time predictions, (2) a larger time-window prediction, as it will allow increasing the available transportation time of a patient to the hospital, and (3) an improved efficacy. Hence, it is highly desirable to develop and apply new methodologies based on advanced signal processing techniques for predicting an SCD event with high accuracy and a good enough time window which allows a patient out of a hospital to arrive and receive timely medical procedures.

In this paper, a new methodology to predict SCD events is presented. The proposal uses as input the patient ECG signal; next, four steps are applied. In step 1, the empirical mode decomposition (EMD) method is used to separate the ECG signal into a set of frequency bands according to the information contained in the ECG signal. In step 2, Katz Index (KI) [43], Higuchi index (HI) [44], Box dimension index (BDI) [45], Shannon entropy index (SEI) [46], and permutation entropy index (PEI) [47] are employed to examine the obtained frequency bands in order to estimate features to be used for SCD prediction. In step 3, the most discriminant features are selected using an analysis of variance (ANOVA). In step 4, the selected features are classified employing a multilayer perceptron (MLP) neural network in order to predict an SCD episode 25 min before the onset in an automatic way. In order to validate the efficiency of the proposal, the MIT-BIH databases of the ECG data measured experimentally from 18 normal and 20 SCD patients are analyzed.

## 2. Methodology

**Figure 1:**
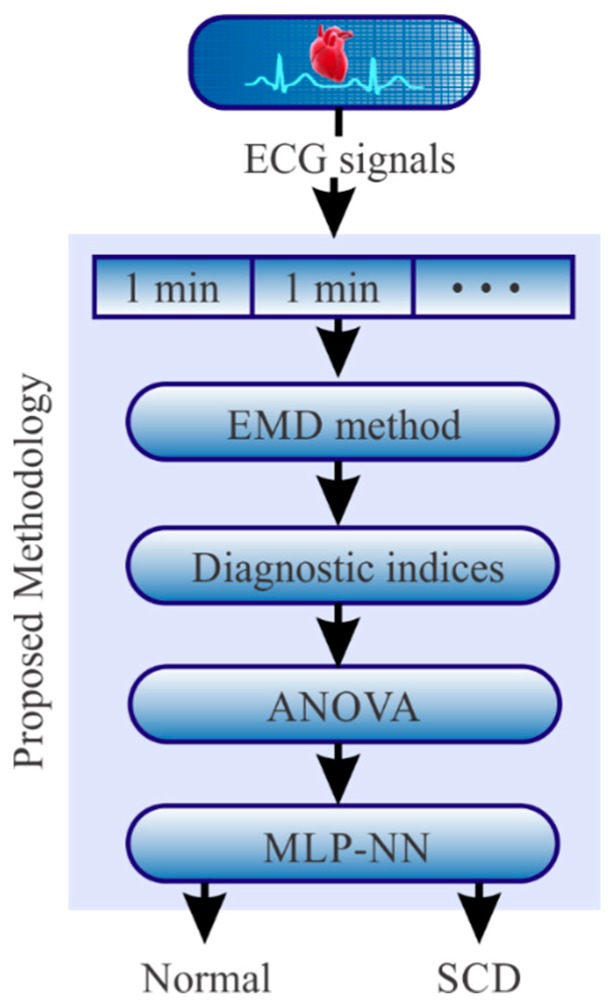
Proposed methodology.

So as to predict the SCD onset, the methodology shown in Figure 1 is proposed. In general, it consists of the following steps. Firstly, the ECG signal is segmented in time windows of 1 min intervals with the aim of applying the proposed methodology for predicting if the signal corresponds to either a normal ECG signal or a signal with an SCD episode. In the next step, once the signal is segmented, the EMD method is applied to each segment in order to decompose it into a set of Intrinsic Mode Functions (IMFs), which can have information or hidden features that contribute to predicting the SCD. To quantify such features in each IMF, the KI, HI, BDI, SEI, and PEI indices are used and tested to determine which one allows distinguishing between a normal condition and an SCD condition. These indices are selected as they have proved to be sensitive to the presence of nonlinear properties in a signal [48,49,50]. Since the EMD method provides different IMFs and all the aforementioned indices are applied to all the IMFs, a great amount of information is generated. In order to reduce this information, the one-way ANOVA statistical method is used since this tool is useful to determine the most discriminatory indices. Finally, the selected indices are used as inputs in a Multilayer Perceptron-Neural Network (MLP-NN) to determine the SCD condition in an automatic way. A detailed description of the mathematical concepts used in the proposal and their application in the processing of ECG signals are presented in the following subsections.

### 2.1. Empirical Mode Decomposition (EMD) Method

The EMD method is a signal processing tool proposed by Huang et al., [51]. This method has been widely used in many research fields because it is capable of processing nonlinear and nonstationary signals [52]. As the features associated with the SCD condition can present nonlinear and/or nonstationary properties, the EMD method becomes a promising tool for their analysis. This method decomposes the signal under analysis in a set of mono-component signals known as IMFs. Huang et al., [51] indicate that an IMF must fulfill two conditions: i) the number of zero crossings and the number of extrema of the decomposed signal have to be either equal or different at most by one, and ii) the average value of the upper and the lower envelopes, defined by the local maxima and the local minima, respectively, is equal to zero. The algorithm to obtain the IMFs is called sifting process and consists of the following steps:

- Step 1: Obtain the extrema points (maxima and minima) of the input signal given by x(t).
- Step 2: Construct the upper and lower envelopes with the maxima and minima, respectively, using cubic-splines.
- Step 3: Compute the mean signal from both envelopes and name it m_1_(t).
- Step 4: Obtain the difference between x(t) and m_1_(t) as h_1_(t) = x(t)-m_1_(t).
- Step 5: Check if h_1_(t) is an IMF (conditions i and ii). If h_1_(t) is not an IMF, steps from 1 to 4 are repeated in a new k-iteration; otherwise, h_k_(t) becomes the first IMF, i.e., IMF_1_.
- Step 6: Obtain the residual signal between x(t) and IMF_1_ as r_1_(t) = x(t)-IMF_1_.
- Step 7: Check if r_1_(t) is a monotonic function, i.e., a signal from which no more IMFs can be extracted. If r_1_(t) is not a monotonic signal, r_1_(t) becomes x(t) and the aforementioned steps are repeated in order to obtain the remaining IMFs. The process ends when r_k_(t) becomes a monotonic function.

At the end, the input signal, *x*(*t*), is decomposed into *N*-IMFs and a final residue, *r_n_*(*t*), as follows:

(1) $$x(t)=\sum_{n=1}^{N}IMF_{n}+r_{N}(t)$$

In the proposal, each 1 min interval of the ECG signal, *x*(*t*), is decomposed in its IMFs, which are analyzed by the proposed diagnostic indices.

### 2.2. Diagnostic Indices

The early and accurate prediction of an SCD requires extracting the nonlinear features of the ECG signal. The EMD splits the ECG information into frequency bands (IMFs) whose need being quantified in order to predict an SCD episode. In this work, several diagnostic indexes such as KI, HI, BDI, SEI, and PEI are tested to select the ones that better suit the requirements of the SCD prediction.

#### 2.2.1. Entropy

In general, entropy attempts to quantify the randomness of a signal [47]. In the proposed methodology, the entropy index could be a useful tool to detect the random behavior in the ECG signal and predict the SCD. Two approximations to the entropy are analyzed.

##### Shannon Entropy Index (SEI)

SEI is a logarithmic value used to indicate how much the measured process changes [46]. In particular, the range of values that SI can take depends on the number of bits used in analog-to-digital converter (ADC). For instance, the SEI for 12 bits ADC ranges from 0 to 12 where a low value, such as DC voltage, means the signal takes few values along time. Conversely, a value near 12 indicates that the signal takes all the possible values of the ADC. In other words, high SEI indicates that all the possible values of the ADC can occur with the same probability. Considering *M* possible values, *v_M_*, for each *IMF_n_* = {*v*_1_, *v*_2_, *v*_3_, ..., *v_M_*} the SEI is calculated by means of Equation (2).

(2) $$SEI_{n}^{}=-\sum_{i=1}^{M}\left[f\left(v_{i}^{}\right)/M\right]\;log_{2}^{}\left(f\left(v_{i}^{}\right)/M\right)$$

where *f*(*v_i_*) is the frequency value *v_i_* of the *IMF_n_*.

##### Permutation Entropy Index (PEI)

As the ECG signal can be considered as a nonlinear time series, the PEI gives a complexity measurement for this type of signal with a high-noise immunity [12]. The normalized PEI quantifies the regularity of the analyzed signal in a range from 0 to 1, where 0 indicates high regularity in the series and 1 indicates the opposite regularity. Defining π as an order pattern of permutations that can occur in the analyzed *IMF_n_*, the PEI is determined by Equation (3).

(3) $$PI_{n}^{}=-\frac{1}{\ln\left(m!\right)}\sum_{i=1}^{m!}p\left(\pi \right)\;\ln\left(p\left(\pi \right)\right)$$

where m is the size of π and *p*(*π*) is the probability that the pattern π occurs in the *IMF_n_*; *p*(*π*) is calculated using Equation (4).

(4) p(π)=f(π)/[K−(m−1)τ]

In this case, *f*(*π*) is the frequency of the pattern π in the *IMF_n_* and τ is the time lag.

### 2.3. Fractal Dimension (FD)

A self-similar signal is considered exactly or approximately similar to a part of itself. FD provides information about the geometrical structure at multiples scales of a self-similar signal [52]. A small FD indicates low complexity in the signal; conversely, a high FD indicates a high complexity in the signal. Then, if the ECG signal is self-similar, FD can provide information about its geometry. Thus, if geometrical changes occur when a person suffers an SCD event, they will be reflected in the FD value, making it a potential index for SCD prediction. Diverse FD methods have been reported in the literature, which can measure the geometrical self-similarities of a signal in a different manner. Hence, in this work three approaches, KI, HI, and BDI, are evaluated to determine if they are capable of identifying patterns to be associated with the rhythm cardiac using ECG signals.

#### 2.3.1. Katz Index (KI)

The KI of a time signal is defined as Equation (5) [43]:

(5) $$KI_{n}^{}=\frac{Log_{10}^{}\left(L_{n}^{}/a\right)}{Log_{10}^{}\left(d/a\right)}$$

where *L_n_* is the total length of the analyzed IMF. This parameter is calculated by means of Equation (6).

(6) $$L_{n}^{}=\sum_{k=2}^{K}distance\left(IMF_{n}^{}\left(k\right)-IMF_{n}^{}\left(k-1\right)\right)$$

In Equation (5), *a* is the average of the total length of the analyzed IMF and is calculated using Equation (7).

(7) $$a=\frac{L_{n}^{}}{K-1}$$

Finally, *d* is the maximum distance between the first value of the IMF and all the possible values of the IMF.

#### 2.3.2. Higuchi Index (HI)

The Higuchi’s FD approach requires being computed in several steps [44]:

- Step 1. Decompose the analyzed IMF into several discrete-time sequences $S_{p}^{q}$
  (8) $$S_{p}^{q}=\left\{IMF\left(q\right),\;IMF\left(q+p\right),IMF\left(q+2p\right),\cdots ,\;IMF\left(q+\left\lfloor \frac{K-q}{p}\right\rfloor p\right)\right\}$$
  where *q* = 1, 2, 3, ..., *Q_max_*, *q* and *p* are integer values which determine the initial IMF value and the number of skipped samples, respectively. *Q_max_* is the maximum number of skipped samples.
- Step 2. Calculate the length of the generated sequences $S_{p}^{q}$ using Equation (9).
  (9) $$L_{q}^{}\left(p\right)=\left\{\left(\sum_{i=1}^{\left\lfloor \frac{K-q}{p}\right\rfloor }\left|S_{p}^{i}-S_{p}^{i-1}\right|\right)\frac{K-1}{\left\lfloor \frac{K-q}{p}\right\rfloor p}\right\}$$
- Step 3. Compute the sum of all the generated sequences by means of Equation (10).
  (10) $$L\left(p\right)=\sum_{q=1}^{p}L_{q}^{}\left(p\right)$$
- Step 4. Calculate the slope of the line that best fits the plotted plane (ln(1/*q*), ln(L(*q*)). The obtained slope is the HI value.

#### 2.3.3. Box Dimension Index (BDI)

The BDI of a time-series signal, in this case, an IMF, is calculated using Equation (11) [45]

(11) $$BDI_{n}^{}=-\frac{\ln\left(\sum_{k=1}^{K-1}\left|IMF_{n}^{}\left(k+1\right)-IMF_{n}^{}\left(k\right)\right|/\Delta t\right)}{\ln\left(\Delta t\right)}$$

where Δ*t* is the sampling-time interval and *K* is the number of samples of the IMF.

### 2.4. The Analysis of Variance (ANOVA)

ANOVA is a statistical tool to analyze sets of quantitative data obtained from various experimental conditions or treatments. One-way ANOVA performs the comparison of two or more statistical means of several populations. The comparison is made by means of a test of hypotheses where a null hypothesis *Ho* is established, the null hypothesis claims that there is no significant difference between the sample means of the analyzed populations [38]. ANOVA generates a test statistic *f*, which determines if the null hypothesis is accepted or rejected at a specific level of significance according to Fisher’s distribution. In statistical packages, the ANOVA generates a table with the analysis of the data, the test statistic, and a probability *p*-value. This *p*-value represents the minimum value of the significance for which the null hypothesis can be rejected. Small *p*-values allow us to easily reject the null hypothesis, indicating that there is a significant difference between the population means of the analyzed data sets.

The ability of ANOVA to differentiate different groups is used in this work to select the entropy and FD indicators that are statistically different for normal individuals and those who presented an SCD. The comparison of the proposed indicators between both groups is done with the objective of reducing the number of indicators, i.e., those that can better characterize both study groups.

### 2.5. Multilayer Perceptron (MLP)

Finally, the most discriminant nonlinear indices that are selected by the ANOVA method are used to train an artificial neural network in order to predict an SCD event in an automatic manner. Neural networks are powerful mathematical tools that simulate models of learning and decision, similar to the behavior of biological brains. The Artificial Neural Networks (ANNs) collect information of various input and output variables, from which the learning and interaction models are built. The input information is processed in different layers of the neural network in order to generate the output values called activation variables. In each layer, there are neurons interconnected by links, in these links there are output values for each neuron, which are multiplied by one weight. The objective of the neural network is to identify patterns and to make decisions on problems that are usually difficult to solve in an analytical way. This tool is widely used in problems of machine learning, pattern recognition and artificial vision, among others [31,53,54]. A multilayer perceptron is a type of feedforward neural network that consists of three main layers: an input layer, one or more hidden layers, and an output layer [52]. This configuration of the MLP allows solving problems that are not linearly separable, capturing the nonlinear relationship in complex data [55].

The output in each neuron is obtained by adding a weighted set of inputs. This sum is defined by Equation (12).

(12) $$S_{j}=\sum_{i=1}^{n}w_{ij}x_{i}+b_{j}$$

where *w_ij_* represents the weighting factors corresponding to the input $x_{i}$ in the layer *j* and *b_j_* is the bias unit added to each neuron. The output *y_j_* is determined by a transfer function *F,* which is defined as:

(13) $$y_{j}=F\left(S_{j}\right)=\frac{1}{1+e^{-S_{j}}}.$$

This transfer function is the sigmoid bipolar. It is employed in this work since it can deal with nonlinear features [52], which can be found in the ECG signals.

## 3. Materials

In order to validate and test the proposal, ECG signals of two international databases are used. The first one is the Normal Sinus Rhythm (NSR) database, MIT/BIH-NSR [41], which includes the ECG signals of 18 subjects. Experts from the Arrhythmia Laboratory at Boston’s Beth Israel Hospital confirmed that the subjects presented a normal heart rate; hence, these signals are taken as ECG signals without properties associated with the SCD condition, i.e., normal condition. The second one is the SCDH database, MIT/BIH-SCDH [42], which includes the ECG signals of 23 subjects with SCD produced by ventricular fibrillation. For these signals, the exact time of the SCD is indicated. This time is used as a reference to predict the appearance of SCD in an earlier way. It is important to mention that only the ECG signals of 18 subjects are used in the analysis because the remaining subjects present other heart alterations such as hypertrophic cardiomyopathy and ventricular tachycardia (future opportunities of research). Table 1 summarizes some features of the used ECG signals; a more detailed description can be found in Amezquita-Sanchez et al., [31]. 

**Figure 2:**
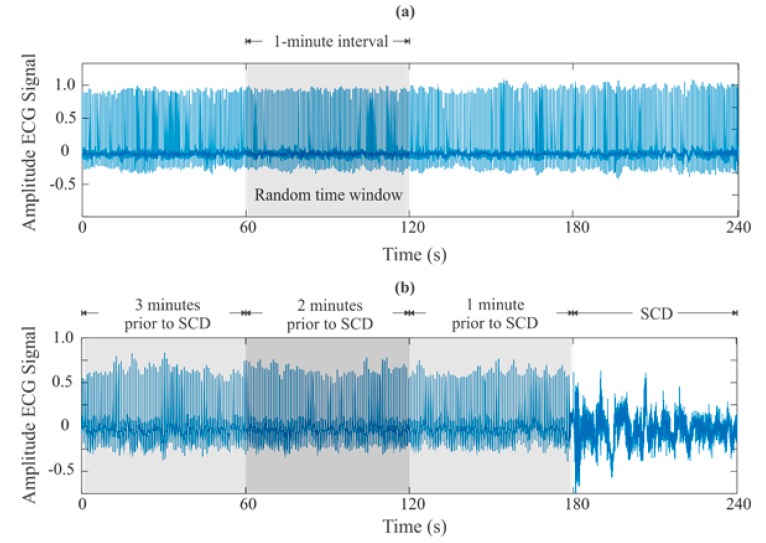
ECG signals for a: (**a**) healthy subject and (**b**) subject with an SCD episode.

As the ECG signals correspond to different databases, some aspects have to be considered. For instance, in the SCDH database (SCD group), a sampling frequency of 250 Hz was used, whereas a sampling frequency of 128 Hz was used in the NSR database (normal group). For consistency, the ECG signals with 250 Hz are down-sampled to 128 Hz. Figure 2 shows the ECG signals for both a person with a normal condition and a person with SCD condition. In these signals, the goal is to detect features that allow predicting the SCD using time windows of 1 min, as used by other authors [36,37,38,39,40]. This window time size is selected to continuously analyze so the patient condition status can be frequently monitored; in this way, medical help can be obtained. Although the ECG signals were monitored for 24 h, only 25 min of them before the SCD occurrences are analyzed. The efforts of this research are put in this time window since it outperforms the reported times by other researchers around the world, providing an earlier SCD prediction. For the ECG signals of the normal group, a 1 min interval of each signal is randomly selected.

## 4. Experimentation and Results

**Figure 3:**
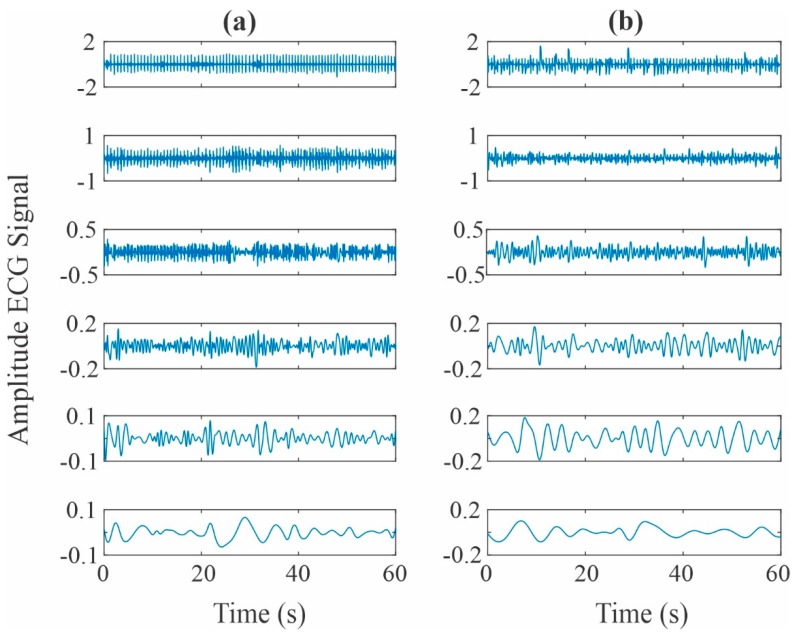
IMF decompositions obtained from an ECG signal of a: (**a**) healthy subject and (**b**) subject with an SCD episode.

Following the steps of the proposed methodology, the 1 min intervals of both the healthy and the SCD group signals are decomposed using the EMD method, where the first six IMFs are used. It should be noticed that the last IMFs are generated due to interpolation errors; thus, they do not contain physical useful information and can be safely discarded for further analysis [56]. To illustrate the analyzed signals, Figure 3 shows the six obtained IMFs for a normal subject and an SCD subject one minute prior to its development. From the figure, it is seen that no visual differences associated with the SCD condition can be detected. In other words, the perceived differences are not produced by the SCD condition [40]; instead, they can be related to the gender, age, or the patient physical condition, among other causes.

After obtaining the IMFs, the five diagnostic indices (KI, HI, BDI, SEI, PEI) are obtained for each IMF. This results in the estimation of 30 indices for each patient and condition. In other words, 480 values are calculated for each 1 min interval. All the obtained values are analyzed using the one-way ANOVA method in order to determine which ones are the most discriminant features. The obtained results show that the PEI value from IMF 5 and HI value from IMF 3 are the ones that have the highest statistical significance, since a low *p*-value is obtained for each index. Consequently, it can be affirmed that both values have the necessary information to distinguish from a person that has a normal heart signal from one that suffers from an SCD episode without considering their age or general physical condition.

**Figure 4:**
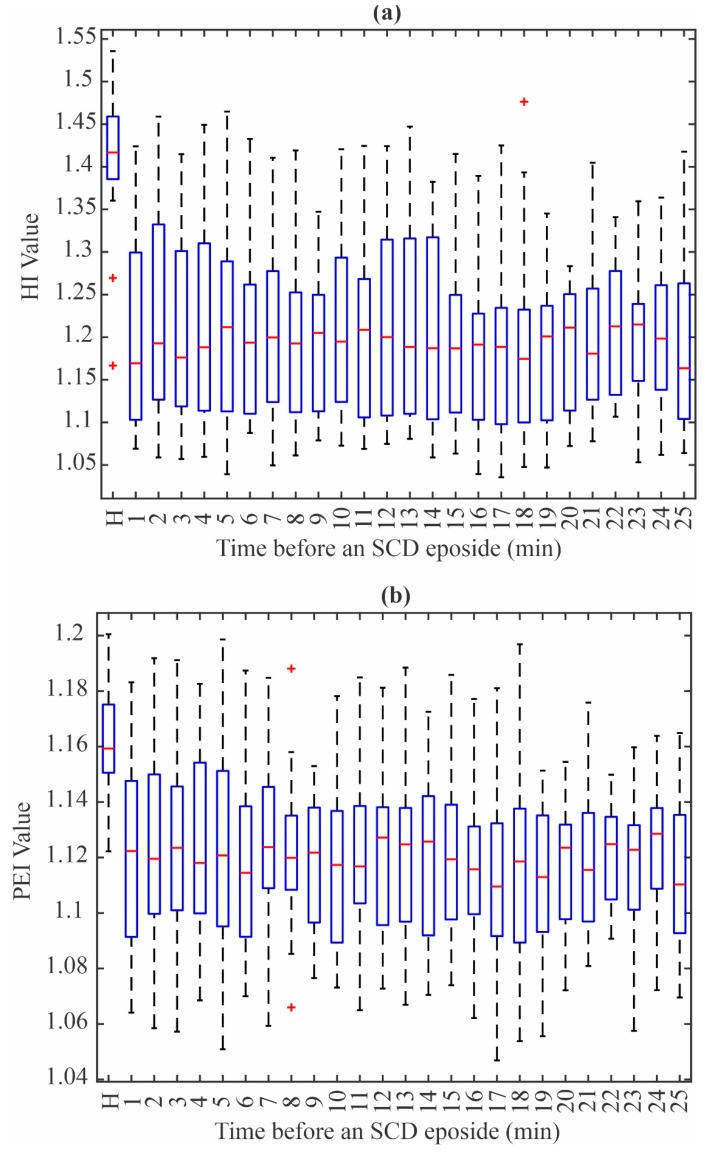
Box plots for (**a**) Higuchi index (HI) from IMF 3 and (**b**) permutation entropy index (PEI) from IMF 5.

Figure 4a,b show the HI value distribution using IMF 3 and the PEI value distribution using IMF 5, respectively. The normal patients are denoted by the letter N, whereas the remaining boxes show the PEI and HI values, respectively, in 1 min intervals up to 25 min before the SCD event. From these figures, it can be seen that there is an evident difference between the values from the N group and the group with an increased risk of suffering an SCD episode. In particular, there are some values that are far from the central zone (denoted by the + symbol), which most of the time fall outside of the zone for normal patients. It should be noticed that the ECG signals can be affected by several factors such as age, body positions, the person’s emotional health, among others [31]. These factors yield to have different cardiac rhythms; thus, different measured waveforms are produced and measured. Nevertheless, the combination of the two indices allows obtaining enough information to generate an accurate classifier.

Table 2 presents a summary of the mean, standard deviation, and *p*-value for the two indices, from 1 min to 25 min before the SCD episode in 1 min intervals. It can be seen that the *p*-value is lower than 0.00001 for all the intervals. After the values are selected (PEI from IMF 5 and HI from IMF 3), an ANN-based classifier is designed. To this purpose, 45% of the available data, i.e., eight patients for each class, were randomly selected and the maximum and minimum values for each 1 min interval of these data were obtained to generate 400 random values within the aforementioned limits. This procedure allows ensuring the necessary diversity to capture all the possible values that can indicate the existence of an SCD event. To obtain the weights and biases, the Levenberg–Marquardt algorithm was employed. Lastly, the classifier was validated using the last 55% of the data, where the patients were also randomly selected. Table 3 shows the accuracy obtained for each interval, where it can be seen that the proposal achieves good accuracy. An average of 94% up to 25 min before the occurrence of an SCD event was obtained with the proposed methodology. This time window allows the patient to take the proper remedial actions (medical treatment), increasing the survival chance.

###  Results Discussion

The obtained results show that an average accuracy of 94% is obtained to predict 25 min before an SCD event occurs, which is a relatively long period of time [57,58,59]. In general terms, it is desirable to have a classifier that can have a 100% accuracy, as this will indicate that both all healthy patients and the ones with an SCD episode are correctly identified; yet, if this situation is not possible, a classifier that has a low number of false-negative detections, i.e., patients that are identified as healthy but they have an SCD episode, is acceptable. On the contrary, if the classifier has a high number of false-negative cases, patients that require immediate medical help will not be identified, increasing the risks of having a life-threatening event. As Amezquita-Sanchez et al., [31] note, the prediction of the SCD event is a challenge as the ECG signal has subtle changes that the diagnostic indices must detect; moreover, it is well known that ECG signals have a nonlinear behavior [31,33,36,39,40]. In this sense, the indices used in this work can properly deal with nonlinear signals [12], which coupled with the ANN classifier allows generating a methodology that can indicate the possibility of having an SCD event in a timely way. Nevertheless, it is important to continue improving the classifier accuracy in order to achieve a 100% accuracy, as this will indicate that neither false-positives (patients that are identified with an SCD episode but are healthy) nor false-negatives are detected, making the proposal a reliable and efficient tool to help physicians in the diagnosis process.

Table 4 presents a comparison between recent articles that uses the same database. The table includes the minutes before the SCD event occurs, the accuracy obtained, and a brief description of the proposed methodology. It can be seen that the proposed methodologies that obtain the highest accuracy values make use of algorithms such as different types of entropies, Hurst exponent, correlation dimension, among others, that can process nonlinear signals; nevertheless, they require the utilization of many indices to perform the prediction, which in consequence increases the required computational time to deliver the result. In contrast, the proposed methodology only uses two indices, which leads to a decrease in the used computational time. On the other hand, it should be noticed that the proposal can predict the likelihood of the SCD event up to 25 min before its appearance, which improves the detection time presented by Amezquita-Sanchez et al., [31] by a margin of 5 min. The development of methods that can expand the detection time of the SCD episodes before its occurrence has been one of the goals of the researchers in this field. Since the detection of the ECG waveform changes before the SCD event increases its difficulty when the time before the SCD episode occur increases; thus, the development of signal processing techniques that can detect those changes also becomes a challenging task. In this sense, the proposal increases 25% the detection time using only two indices, which is a reasonable compromise to allow performing an on-line operation. It should be noticed that the proposal obtains the features used for classification from the raw ECG signal, unlike other methodologies that make use of the HRV signal, which increases the computational time required to execute the proposed methodologies, as they have to use further signal processing strategies to correctly detect the heartbeats required to estimate the aforementioned signal [60].

## 5. Conclusions

The existence of an SCD event generates subtle changes in the ECG signal that can be undetected due to the contained noise in the measured signal. This feature makes SCD detection a challenging task. Because of this, the earlier the detection, the better the possibility of surviving the event. This paper proposes a new methodology that combines EMD, nonlinear indices, and neural networks to predict the occurrence of an SCD event 25 min prior to the onset, using ECG signals from the MIT/BIH-SCDH and the MIT/BIH-NSR databases [41,42]. The obtained results show that the HI and PEI values of the IMF 3 and IMF 5 can be used to detect the event, as the one-way ANOVA values show that they have the most relevance. With this information, an ANN classifier was developed, achieving a 94% accuracy. Moreover, the prediction time window of an SCD event is improved by 25% and is compared with the proposal that has the largest time window [31], giving additional time to receive medical assistance and increasing the possibilities of overcoming the event without severe physical consequences.

As future work, the proposed method will be tested using a larger database and smaller time windows in order to prove the consistency of the results; also, the assessment of the efficacy of different classifiers could lead to an increase of the prediction time can lead to further improve the possibilities of mitigating the medical effects that an SCD event can have.

## Figures and Tables

**Table 1:** Information about sudden cardiac death (SCD) and normal databases.

Database	Group	Subjects (Sex)	Age
NSR	Normal	18 (13 female)	35 ± 15
SCDH	SCD	23 (8 female)	49.5 ± 32.5

**Table 2:** Information about the SCD and normal databases.

Normal			SCD		
Fractal	μ ± σ	Minute	Fractal	μ ± σ	*p*-Value
HI	1.4061 ± 0.0892	25	HI	1.1875 ± 0.1077	4.71 × 10^−07^
PEI	1.0793 ± 0.0143		PEI	1.0505 ± 0.0126	1.25 × 10^−06^
		24	HI	1.2055 ± 0.1231	3.77 × 10^−07^
			PEI	1.0556 ± 0.0138	4.42 × 10^−05^
		23	HI	1.2014 ± 0.1187	1.71 × 10^−07^
			PEI	1.0533 ± 0.0128	7.37 × 10^−06^
		22	HI	1.2050 ± 0.1082	2.16 × 10^−07^
			PEI	1.0528 ± 0.0113	2.28 × 10^−06^
		21	HI	1.1948 ± 0.1051	1.02 × 10^−06^
			PEI	1.0517 ± 0.0110	1.02 × 10^−06^
		20	HI	1.1885 ± 0.1038	2.31 × 10^−08^
			PEI	1.0539 ± 0.0119	6.28 × 10^−06^
		19	HI	1.1829 ± 0.0874	8.24 × 10^−08^
			PEI	1.0498 ± 0.0114	4.11 × 10^−07^
		18	HI	1.1943 ± 0.1167	5.04 × 10^−06^
			PEI	1.0507 ± 0.0191	4.19 × 10^−05^
		17	HI	1.1915 ± 0.1065	1.50 × 10^−06^
			PEI	1.0507 ± 0.0163	1.09 × 10^−05^
		16	HI	1.1902 ± 0.1173	8.04 × 10^−07^
			PEI	1.0521 ± 0.0166	2.51 × 10^−05^
		15	HI	1.1925 ± 0.1270	6.35 × 10^−07^
			PEI	1.0527 ± 0.0136	7.55 × 10^−06^
		14	HI	1.2034 ± 0.1191	6.55 × 10^−06^
			PEI	1.0511 ± 0.0151	7.00 × 10^−06^
		13	HI	1.2160 ± 0.1026	3.07 × 10^−05^
			PEI	1.0550 ± 0.0147	4.98 × 10^−05^
		12	HI	1.2165 ± 0.1071	1.54 × 10^−05^
			PEI	1.0544 ± 0.0146	3.42 × 10^−05^
		11	HI	1.2086 ± 0.1126	3.37 × 10^−06^
			PEI	1.0516 ± 0.0165	1.92 × 10^−05^
		10	HI	1.2160 ± 0.1241	1.42 × 10^−05^
			PEI	1.0531 ±0.0172	5.66 × 10^−05^
		9	HI	1.1960 ± 0.0905	1.85 × 10^−07^
			PEI	1.0527 ± 0.0125	4.16 × 10^−06^
		8	HI	1.2052 ± 0.0742	2.01 × 10^−06^
			PEI	1.0549 ± 0.0180	8.71 × 10^−05^
		7	HI	1.2114 ± 0.0923	3.75 × 10^−06^
			PEI	1.0557 ± 0.0164	8.92 × 10^−05^
		6	HI	1.2039 ± 0.0811	2.66 × 10^−06^
			PEI	1.0524 ± 0.0156	1.88 × 10^−05^
		5	HI	1.2076 ± 0.0819	8.69 × 10^−06^
			PEI	1.0541 ± 0.0184	1.55 × 10^−04^
		4	HI	1.2144 ± 0.0861	2.07 × 10^−05^
			PEI	1.0547 ± 0.0172	1.21 × 10^−04^
		3	HI	1.2052 ± 0.1038	2.85 × 10^−06^
			PEI	1.0546 ± 0.0184	9.37 × 10^−05^
		2	HI	1.2192 ± 0.1194	2.23 × 10^−05^
			PEI	1.0553 ± 0.0197	9.24 × 10^−04^
		1	HI	1.2019 ± 0.1108	2.87 × 10^−06^
			PEI	1.0530 ± 0.0168	4.29 × 10^−05^

**Table 3:** Obtained accuracy for each 1 min interval.

Minute	Accuracy
1	90%
2	100%
3	90%
4	100%
5	90%
6	100%
7	90%
8	100%
9	100%
10	90%
11	90%
12	90%
13	100%
14	90%
15	100%
16	90%
17	90%
18	100%
19	90%
20	100%
21	100%
22	90%
23	90%
24	100%
25	100%

**Table 4:** Comparison with similar works.

Author	Signal	Methodology	Prediction Time (Accuracy)
Shen et al., (2007) [61]	ECG	Three features used as factors to predict the SCD are: (1) HRV mean, (2) the ratio Low frequency/High frequency, and (3) Very low frequency. An artificial neural network is used as classifier.	2 min (67.44%)
Ebrahimzadeh and Pooyan (2011) [62]	HRV	Three analyses are carried out. The first one uses features from both the time and frequency-domain. The second one is a TF domain analysis, in which 10 features are extracted (maximum energy, minimum energy, the difference between maximum and minimum in each window, standard deviation between energy of time windows, the total and average energy of signal in the very low frequency (VLF), low frequency (LF), and high frequency (HF) bands. The last analysis, a nonlinear one, uses two different parameters from the RR intervals: (1) Poincaré and (2) Detrended fluctuation analysis (DFA). The K-nearest neighbor is used as classifier.	4 min (83.96%)
Ebrahimzadeh et al., (2014)	HRV	5 features in time-domain are extracted: mean of all NN intervals (MNN), standard deviation of all NN intervals (SDNN), the square root of the mean of the sum of the squares of differences between adjacent NN Intervals (RMSSD), the standard deviation of differences between adjacent NN intervals (SDSD), the proportion derived by dividing the number of interval differences of NN intervals greater than 50 ms by the total number of NN intervals (PNN50). 4 features in frequency-domain (VLF, LF, HF and the ratio LF/HF) are also employed. Principal component analysis is used to select the most relevant features. An MLP is used as classifier.	1 min (99.16%)
Acharya et al., (2015)	ECG	18 nonlinear features are extracted by using different methods such as Dimension fractal (FD), Hurst exponent (H), approximate entropy (ApproxEnt), Sample entropy (SampEnt), Detrended fluctuation analysis (DFA), and Correlation dimension (CD). Decision tree (DT), Support vector machine (SVM) and K-Nearest Neighbor are the classifiers used.	4 min (92.11%)
Fujita et al., (2016)	HRV	HRV signals are extracted by using the Pan-Tompinks algorithm. Signals are denoised using a DWT-based scheme. From each interval, 12 features are estimated: Fuzzy entropy (FE), Renyi entropy (REnt), Hjorth parameters, Tsallis entropy (Tent), and the energy obtained from the 3-level decomposition, i.e., 8 energy values.	4 min (94.7%)
Amezquita-Sanchez et al., (2018)	ECG	min interval signals are decomposed using the wavelet packet transform (WPT) to generate uniform frequency bands. The homogeneity index (HI) is used as feature. The classification is carried out using the Enhanced Probabilistic Neural Network (EPNN).	20 min (95.8%)
This work	ECG	EMD is used to obtain the signal components (IMFs). Higuchi Fractal value and Permutation Entropy are used as features. MLP is the classifier	25 min (94%)
